# Effects of an Active Visuomotor Steering Task on Covert Attention

**DOI:** 10.16910/jemr.12.3.1

**Published:** 2019-08-08

**Authors:** Samuel Tuhkanen, Jami Pekkanen, Esko Lehtonen, Otto Lappi

**Affiliations:** University of Helsinki, Finland; University of Leeds, United Kingdom; VTT Technical Research Centre of Finland, Finland

**Keywords:** Covert attention, driving, eye tracking, gaze, peripheral vision, Posner cue paradigm, steering

## Abstract

In complex dynamic tasks such as driving it is essential to be aware of potentially important targets in peripheral vision. While eye tracking methods in various driving tasks have provided much information about drivers’ gaze strategies, these methods only inform about overt attention and provide limited grounds to assess hypotheses concerning covert attention. We adapted the Posner cue paradigm to a dynamic steering task in a driving simulator. The participants were instructed to report the presence of peripheral targets while their gaze was fixed to the road. We aimed to see whether and how the active steering task and complex visual stimulus might affect directing covert attention to the visual periphery. In a control condition, the detection task was performed without a visual scene and active steering. Detection performance in bends was better in the control task compared to corresponding performance in the steering task, indicating that active steering and the complex visual scene affected the ability to distribute covert attention. Lower targets were discriminated slower than targets at the level of the fixation circle in both conditions. We did not observe higher discriminability for on-road targets. The results may be accounted for by either bottom-up optic flow biasing of attention, or top-down saccade planning.

## Introduction

Moving in complex environments, whether by walking, bicycling or driving, is a challenging task, widely engaging perceptual, cognitive and motor processes. These include selective attention to multiple targets (1), active visual sampling to pick up relevant information (2, 3), as well as planning and executing the lateral and longitudinal control movements themselves (4, 5).

Typically, we direct gaze to locations we selectively attend to. While steering a path, we generally look where we are going, and go where we look (6, 7). Covert attention, on the other hand, refers to the ability to selectively attend to targets in the visual periphery without overtly directing gaze at them (8, 9). Because of locomotor-task-relevant limitations on visual processing in the periphery (10), it is often more efficient to direct gaze to the relevant visual targets at the appropriate time. Yet in dynamic tasks it is not always feasible to serially go through to all relevant targets with overt gaze: saccadic gaze shifts take time during which visual blurring and saccadic suppression render the subject relatively “blind”. Also, during the ensuing stable fixation, the new visual information does not arrive instantly and takes time to process, as does the programming of the next saccade. Thus, the frequency of saccades cannot be increased and fixation duration reduced without limit; as more targets need to be attended to, it becomes more and more necessary to rely on peripheral vision as one can not serially scan everything in time with overt eye movements. And of course, the decision of where to make the next saccade always has to be made in part on the basis of peripheral vision. In fact, according to the premotor theory of attention (11, 12) the same visuomotor mechanisms underlie saccade planning and a shift of (covert) attention to a target in the visual periphery.

Eye tracking methods in naturalistic and simulated driving tasks have provided a great deal of information about drivers’ visual behaviours and spontaneous gaze strategies (13, 14, 15, 16, 17). It is known from previous research that the majority of fixations whilst driving fall on the road, a few seconds “into the future” (guiding fixations with about 2s time headway, 7, 16, 18) with intermittent look-ahead fixations further up the road (16, 19, 20). For experienced drivers, this is a highly automatized process. The visual information presumably allows for the kind of anticipatory steering control that has been posited by various steering models (20, 21, 22). If redirecting visual attention relies on the same motor programming and dynamic sensory remapping processes as overt (saccadic) gaze shifts, and drivers frequently shift between guiding and look-ahead fixations, all else being equal, it may be expected that more attention would be directed to locations where anticipatory saccades typically land even at an equal distance from the point of fixation. Conversely, if saccade planning and shifts of covert attention use the same mechanisms, then natural visual strategies of dynamic tasks can inform about likely covert attentional shifts even in the absence of overt eye movements.

In a different line of research, it has been shown that adding (radial) visual flow to naturalistic scene displays can capture or direct attention toward the focus of expansion: Wang, Fukuchi, Koch and Tsuchiya (23) found that attention (measured by speeded discrimination) was attracted to a singularity in coherent image motion similar to the focus of expansion which during linear self-motion specifies direction of current heading (more so in “zooming in” to a naturalistic image than for a random dot flow field). When traveling on a curved path, however, the flow field is more complex and lacks a focus of expansion (24), so it is not immediately clear how this result should generalize to locomotion on winding paths. Analogously to the simpler radial case, one might, however, argue that the coherent local visual motion in the flow field would attract (covert) attention up and into the direction of rotation (“against the flow lines” as it were).

### Aims of the study

In the original Posner cue paradigm (8), visual stimuli are presented on opposite sides of a screen. Attention and gaze are decoupled by instructing the participant to maintain gaze on a central fixation target (cross or arrow), and to respond as quickly as possible when they detect a target in the periphery. Covert attention can be cued by making the central fixation target a directed arrow that reliably (80%) indicates which side of the screen the target will appear in. Reaction times for valid-cue targets are observed to be lower than invalid-cue targets.

Our goal was to see whether and how the complex visual stimulus (such as optic flow generated by self-motion) and active visuomotor steering control might affect directing covert attention to visual stimuli in the periphery. We developed a covert attention discrimination task inspired by the classical Posner cue paradigm, where covert attention could be indexed by peripheral visual discrimination in a dynamic steering task, where the discrimination targets would be embedded in a more complex continuous stream of information more characteristic of natural tasks. We reasoned that in the steering task, planned eye movements (look-ahead fixations), and/or planned steering movements (in the direction of the bend) and/or visual flow (down and against the direction of the bend) could analogously “cue” covert attention.

## Methods

### Participants

Twenty-six (26) subjects participated in the study (12M, 14F). Age varied between 21-38 years (mean 29 y, standard deviation 5.7 y). Participants were recruited through university student organization mailing lists and personal contacts. Two sports/culture vouchers of 5 € were offered as compensation.

Participants were required to have a valid driver’s licence, and a minimum of 20 000 km lifetime car driving / motorcycle riding experience, and reported normal vision or corrected-to-normal vision (in which case the participant would wear contact lenses). They reported no known neurological or ophthalmological conditions. All participants were naïve as to the research question and hypotheses.

### Equipment

**Figure 1:**
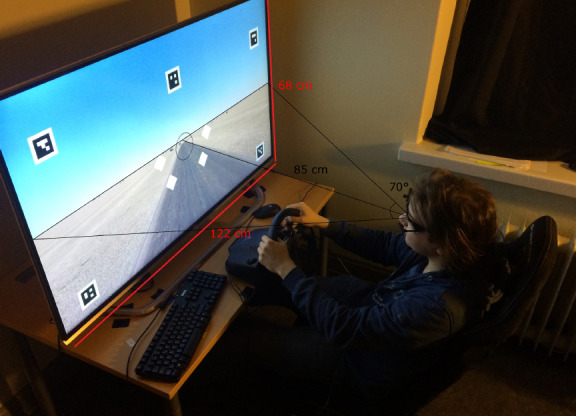
The experimental set-up. During the experiment, the display was the only light source.

Participants sat in a Playseat Evolution gaming chair and controlled the virtual car using a Logitech G26 steering wheel (see Figure 1). Pedals were not used as all participants drove at the same, fixed, speed (80 km/h). The driving simulation was custom built by the research team (JP, ST), and the source code is available at: https://github.com/samtuhka/webtrajsim/tree/graduVersio

The simulation was run and eye tracking data recorded on an ASUS UX303L laptop, running the Linux based Ubuntu (kernel 3.19) operating system. The display used was an LG 55UF85 55” LCD monitor, with the resolution set to 1920 x 1080 px and refresh rate 60 Hz. At the typical 85 cm viewing distance, the screen subtended a visual angle of 70° (which was used as the value for the field of view of the virtual camera in the simulation).

The eye tracker used was Pupil Labs High Speed Binocular (Pupil Labs UG haftungsbeschränkt, Berlin, Germany), and the calibration and data collection was done using the open source Pupil Capture software with custom modifications (available at: https://github.com/samtuhka/pupil), such as modified placement of calibration dots (16 calibration dots) so that they would not be displayed behind the steering wheel, and slightly altered pupil detection parameters.

The maximum sampling frequency of the eye cameras is 120 Hz at 640 x 480 Hz resolution, and the maximum sampling frequency of the forward-looking world camera is 60 Hz at 1280 x 720 px - however, only 30 Hz was used for both in the experiment because the same computer needed to run data collection and simulation.

### Stimuli, Procedure & Measurement

Upon arrival at the lab, the participants signed an informed consent form explaining the general purpose of the experiment. Also, general background information was collected (age, sex, driving experience, gaming experience generally, and driving game experience specifically).

The eye tracker was calibrated by asking the participant to look at visual markers presented successively on the screen. Then the participant drove one practice run on the steering task, and one trial on the control task (see below). Practice run data was not analyzed. The eye tracker calibration was verified, and then the participant performed four steering (S) and four control (C) tasks in blocks of two (half of the participants SSCCSSCC, half CCSSCCSS). The experimenter (ST) sat in the back of the laboratory monitoring the gaze signal from another monitor not visible to the participant. The eye tracker was recalibrated between runs if the experimenter deemed that the gaze position had deteriorated (e.g. due to the movement of the headset). Calibration accuracy was verified at the end of the experiment by asking the participant to look at markers displayed on the screen.

The experiment in its entirety took approximately one hour, after which the participants were debriefed about the purpose of the experiment.

### Steering task

**Figure 2:**
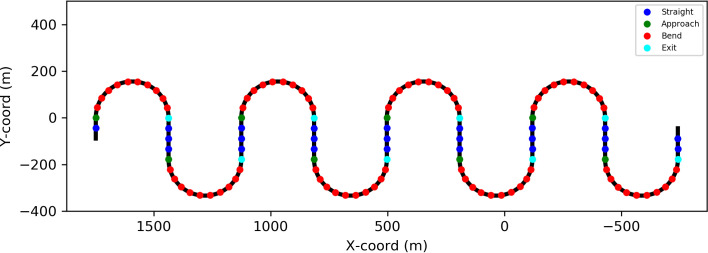
Bird’s-eye view of the track layout. Dots indicate the locations where the targets and distractors were displayed. Blue dots indicate presentation on the straights, red dots during steady-state cornering, and green and cyan dots the transitional entry and exit phases (which were not analyzed due to the small number of observations).

The participants drove a virtual car along a winding road (Figure 2). The track consisted of straight segments (178 m, about 8s) and semi-circular bends (radius 155,6 m, circumference 489 m, about 22s). Lane width was 3.5 m. Total track length for one run was 5 333 m (about 4 min). Engine noise was played through the monitor loudspeakers. In terms of steering, the participants were simply instructed to stay within the lane (instructions were presented on the screen). There was a warning sound (a ‘beep’) to indicate the participant if they had veered off the lane – this also signaled the experimenter in the back of the laboratory if the participant had trouble performing the task.

The virtual ground was flat, and the textures resembled an asphalted road in a sand desert. Rocks were rendered in the scene to enhance the sense of scale, depth and motion.

Participants were instructed to keep gaze within a fixation circle (radius 2.75°), which was placed in the middle of the road at a two-second time headway (44.4m at 80 km/h). The participants were told that the eye-tracker would register it if they directed their gaze outside the fixation circle (but there was no clear prioritization given between the gaze and steering instructions – participants were simply told to do both). A fixation circle was used instead of a fixation cross so as not to abolish the natural optokinetic nystagmus eye movements observed in curve driving (25, 26). A two second time headway corresponds to the typical time headway of “guiding fixations” in driving (16). This places the centre of the circle in the “far” region a few degrees below the horizon (cf. 22). Four white diamond-shaped target areas (edge length 2.5°) were placed radially in the lower visual field, at 7.5° distance from the centre of the fixation circle.

**Figure 3:**
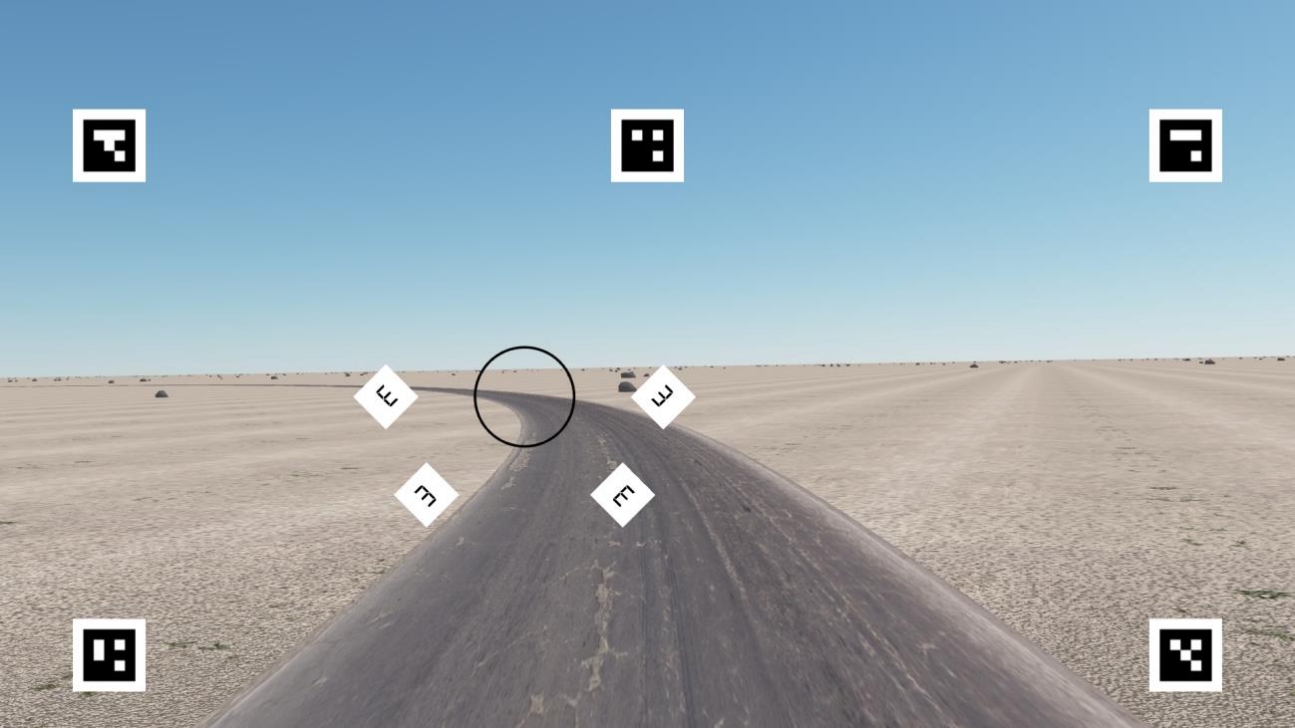
Screen capture from the steering task, showing the fixation circle, target locations (white diamonds) surrounding it, with a target (in the HI-BEND position) and three distractors. The square screen markers around the edges were used to detect the screen from the image of the eye tracker's front camera and determine a transformation from the camera coordinates to the screen coordinates.

The placement of the targets was chosen so that they would fall in as natural positions as possible with respect to the underlying road scene. See Figure 3. The white target area was visible all the time. The reason for this was to prevent the optic flow from masking the targets. The target and distractor shapes were E symbols akin to those used in standard visual acuity tests. Considerable piloting was used to select target shapes that would be discriminable, but not trivially so (ruling out e.g. red targets and green distractors that would lead immediate pop-out and faint, Gabor patches that turned out to be too difficult). The E letter discrimination was also hoped to be semantically familiar and therefore less artificial to participants used to typical visual acuity tests.

The participant was instructed (instructions were presented on the computer screen) to press the right-hand gear selector paddle behind the wheel whenever they detected an E open up and to the right, and disregard the other shapes.

The targets/distractors appeared within the target areas at fixed 44.4 m (2s) intervals and were visible for 0.5 s. Even after the targets had disappeared, the participant had another 1.5 s to press the lever until the appearance of the next set of targets/distractors, which would be considered a valid detection. This was explained to the participant.

The probability of target appearance during any presentation was 50%, and during each presentation, a target would be present in at most one target location. The target never appeared in the same location on successive presentations, but the locations were otherwise random.

For analysis, the runs were segmented into four phases (see figure 2): straight, turning into a bend, cornering in a bend, exiting a bend. Entry was defined as the +/- 2s from the geometrical beginning of the bend, and exit as the +/- 2s from the geometrical endpoint. While theoretically interesting, these brief transitional events were not analyzed further, as there is only sufficient data in the longer segments (straights and bends). Over the four trials, on straights, the target appeared in each location on average 12 times (there were 96 presentations in total), and 40 times (320 presentations in total) in bends.

### Control task

**Figure 4:**
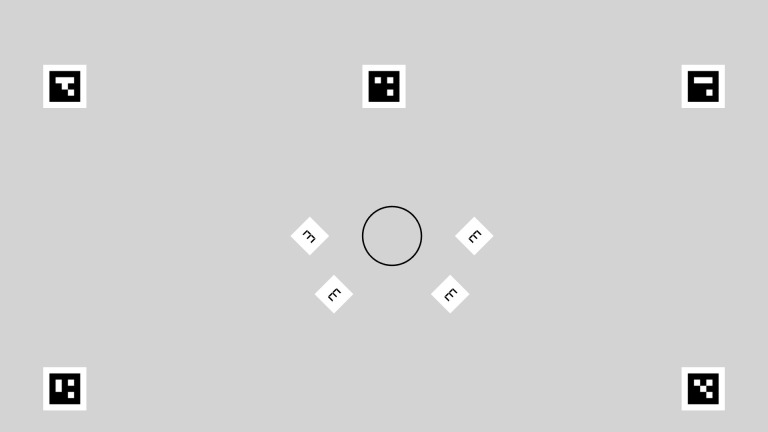
Screen capture from the control task. The fixation circle and target presentation were the same as in the steering task, but the road view was not visible and the participant did not actively steer. The horizontal movement of the fixation circle and target position display corresponded to the movement produced by a path following the road centre (in fact movement of the vehicle was simulated behind the scenes). The square screen markers around the edges were used to detect the screen from the image of the eye tracker's front camera and determine a transformation from the camera coordinates to the screen coordinates.

The same task was used in the control condition as in the steering condition, except that the road was not displayed (see Figure 4), and the participants did not actively steer. (The virtual car drove on autopilot so that the array formed by the fixation circle and target areas moved horizontally as in the steering task). No engine noise was played. The number of trials and target presentations was identical to the steering task. The instructions for the participants were otherwise the same as in the steering task but there was no mention of steering.

### Post-processing and analysis

Post-processing was done using custom Python scripts using the SciPy, NumPy and matplotlib libraries (analysis source code available at: https://github.com/samtuhka/gazesim_tools).

Barrel distortion of the world-camera image was corrected. Gaze coordinates were transformed into undistorted screen coordinates using localization markers in the screen edges (produced in the simulation). Eye tracker data was synchronized with simulator events using Unix timestamps. Offline calibration was done by polynomial regression between calibration dots algorithmically identified from the world camera image and pupil location in eye camera image during calibration.

**Figure 5:**
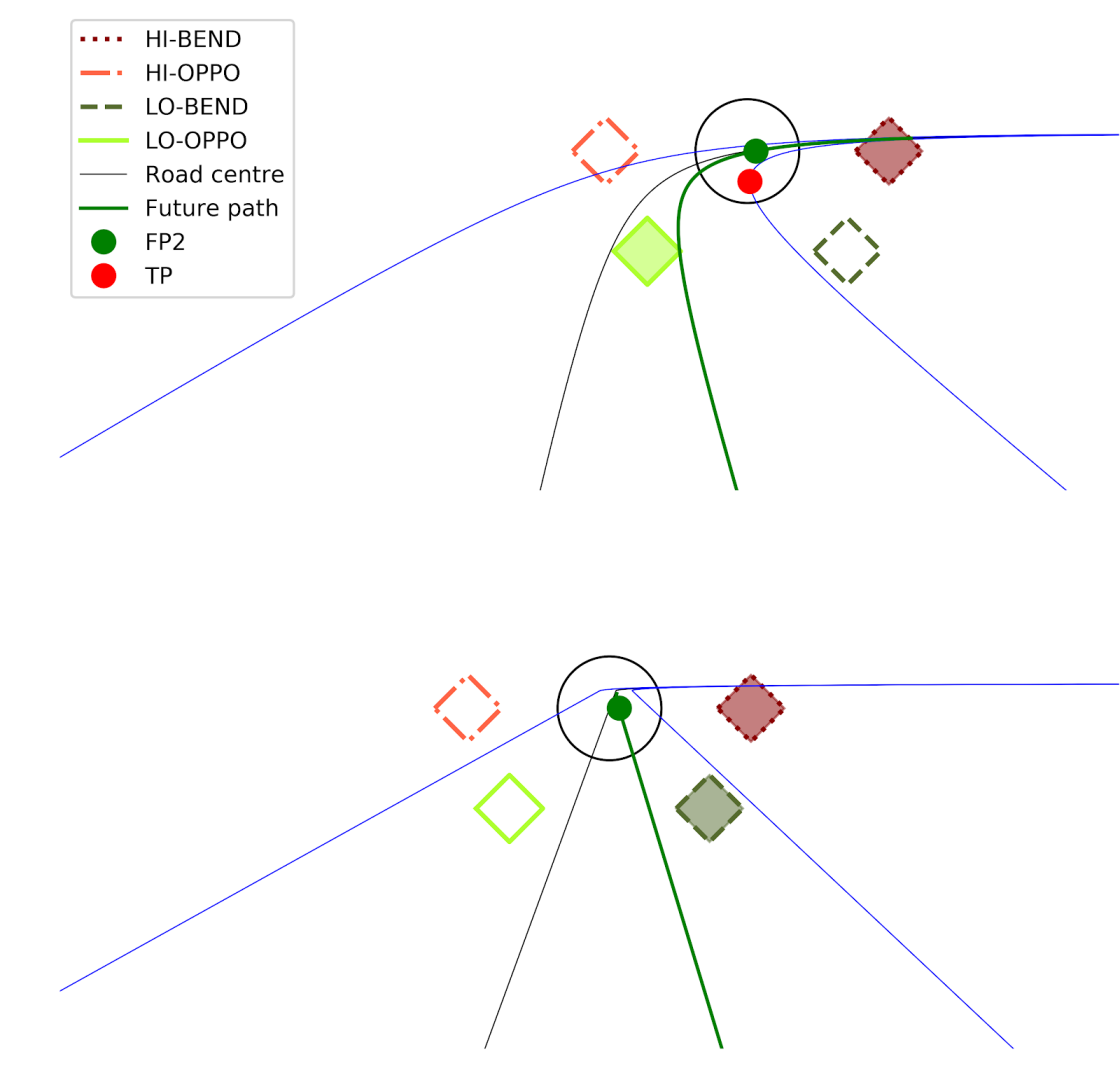
*Top*. The distribution of gaze points in relation to the fixation circle and the different target locations during target presentations that were accepted into the analysis in the steering task. If a single gaze point during a target/distractor presentation was outside of the blue region of interest (ROI), the presentation was excluded from the analysis. *Bottom*. Otherwise the same distribution, but for the control task.

Calibration accuracy evaluated on basis of the final verification was estimated to be about 1° (mean 1.02°, standard deviation 0.67°) on the horizontal axis and about 2° (mean 2°, standard deviation 1.47°) on the vertical axis. (Calibration accuracy immediately after calibration mean 0.55°, standard deviation 0.36° horizontal, 0.42° vertical). Three subjects showed significant constant bias due to the movement of the headset, which was corrected on the basis of the final verification. For each target/distractor presentation, cases, where the participant’s gaze had not remained within the region of interest around the fixation circle, were rejected (see Figure 5). Three participants’ data was removed due to poor eye tracking data quality or excessive (>25%) fixations outside the designated fixation area (i.e. 23 subjects were included in the final analysis). The gaze distribution from the target presentations that were accepted into the analysis can be seen in Figure 5.

Possible variability in steering performance was not analyzed in depth, but participants were observed to have little to no trouble in performing the steering task. Though the speed was comparable to driving on a highway (80 km/h), the radius of the bend was quite high (155,6 m) making the required yaw rate relatively low (~8°/s). In total, the participants spent an average of 0.34 seconds (SD = 1.04 s, range = 0–4.9 s) outside of the lane boundaries during the four steering trials.

**Figure 6:**
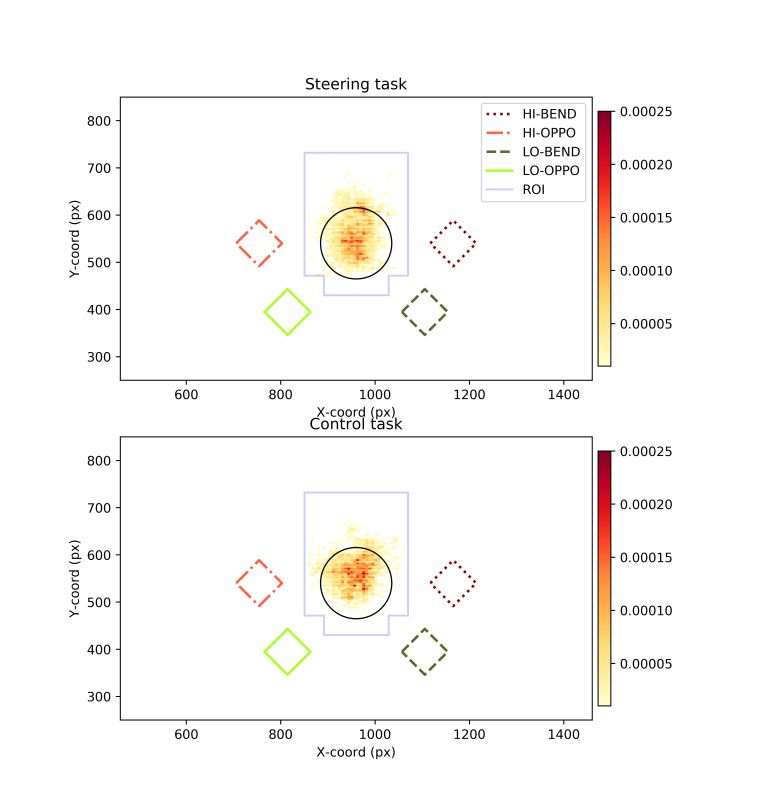
Target positions in bends (top panel) and on straights (bottom panel). Green dot (FP2) = future path travel point at 2s time headway; Red dot (TP) = Tangent point (bend only). The shaded targets mark the locations where based on prior literature we expected better detection performance. If planned but not executed look-ahead fixations produce a top-down attentional cueing effect, then the detection of targets presented in the HI-BEND location should be facilitated compared to the detection of targets in the HI-OPPO location. If the horizontal optic flow during cornering leads to a bottom-up attentional cueing effect, this should be observed in the bends (only). While LO-BEND falls closer to the road edge than LO-OPPO, in bends it typically falls outside the road whereas LO-OPPO is situated close to the future-path. If attention is spread evenly along the future path, then both the HI-BEND and (in corners) LO-OPPO target detection should be facilitated.

The four target locations were placed radially symmetrically in the lower visual field, around the fixation circle placed at a time headway of 2 seconds which corresponds to the normal guiding fixation preview distance (16). The tangent point (14) also falls within the fixation circle. They were classified as shown in Figure 6 (bend direction vs. opposite to bend direction and up vs. down). On the basis of theoretical considerations and prior experimental literature, on both straights and in the bends the target up and in the bend direction (HI-BEND) was expected to be the most attended one (it is in the look-ahead fixation region of the visual field, i.e. would be target of gaze polling saccades from guiding to look-ahead fixations, if they were not disallowed by the task instruction; 16, 25). The target down and in the direction of the bend (LO-BEND) is near the road edge (which is considered to be peripherally monitored in driving; 27, 28, 29). On a straight, neither of the down targets falls on the road substantially more than the other, but in bends, the down target opposite to the direction of the bend (LO-OPPO) more clearly falls on the future path (Figure 6).

## Results

### Peripheral target detection

**Figure 7:**
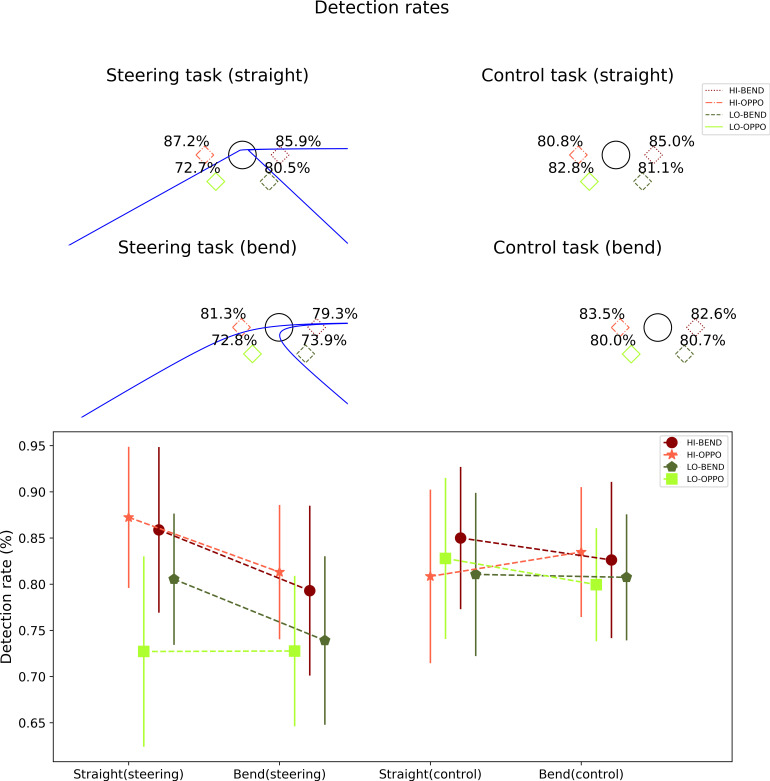
*Top*. Mean detection ratios for targets presented in the different target locations, in the steering and control tasks and on the straights and in the bends. *Bottom*. The mean detection rates and standard deviations in a line plot.

Target detection was investigated by calculating the ratio of reported identifications (when there was a target present) and the number of target presentations at that position. We call this variable the detection ratio which we report as a percentage. It was calculated separately for each target position, on the straights and in the bends, in both the steering and the control task (Figure 7 and Table 1). Please note that because the response for detecting a target was always the same irrespective of the target position, it is not possible to calculate position-wise false positive ratios. However, the overall rate of false positives was negligible (mean 1.3%, standard deviation 1.5%).

**Table 1:** Mean detection rates and standard deviations for the different target locations in straights and bends in both the steering and control tasks.

Steering task (straight)	HI-BEND	HI-OPPO	LO-BEND	LO-OPPO
Mean	85.5%	87.2%	80.5%	72.7%
SD	17.7%	15.3%	14.2%	20.6%
Steering task (bend)	HI-BEND	HI-OPPO	LO-BEND	LO-OPPO
Mean	79.3%	81.3%	73.9%	72.8%
SD	18.4%	14.5%	18.2%	16.3%
Control task (straight)	HI-BEND	HI-OPPO	LO-BEND	LO-OPPO
Mean	85.0%	80.8%	81.1%	82.8%
SD	15.4%	18.8%	17.7%	17.4%
Control task (bend)	HI-BEND	HI-OPPO	LO-BEND	LO-OPPO

By subtracting the control task detection ratios from the corresponding steering task detection ratios, we get detection ratio changes (Table 2), which can be used to assess the overall and location-specific effect of the complex (flow) stimulus and active steering task on peripheral detection.

**Table 2:** Detection rate changes and standard deviations for the different target locations in straights and bends.

Normalized (straight)	HI-BEND	HI-OPPO	LO-BEND	LO-OPPO
Mean	0.5%	6.4%	−0.5%	−10.1%
SD	14.7%	13.2%	16.6%	21.9%
Normalized (bend)	HI-BEND	HI-OPPO	LO-BEND	LO-OPPO
Mean	−3.3%	−2.2%	−6.8%	−7.2%
SD	14.6%	8.3%	11.7%	12.2%

Differences in detection ratio changes across target locations were statistically different at *p* < 0.05 significance level on the straights (Friedman test *Q* = 8.65, *p* = 0.03), but not in the bends (Friedman test *Q* = 2.11, *p* = 0.55). As the detection ratio values did not appear to be normally distributed, the differences between locations in detection ratio change on the straights were investigated using Wilcoxon signed rank test with Bonferroni correction (Table 3). Bi-serial rank correlation *r* was estimated using Kerby’s simple difference formula (30). Pairwise comparisons indicated that detection of targets in the LO-OPPO target position was impaired in the steering task significantly more than detection of targets in the HI-OPPO position (table 3). The other comparisons were not significant.

**Table 3:** Pairwise comparisons of the hit rates for different target positions while driving on a straight.

HI-BEND	HI-OPPO	LO-BEND	LO-OPPO
HI-BEND	*T*= 69.5 , *p*= 1.0, *r*= -0.34	*T*= 124, *p*= 1.0, *r*= 0.02	*T*= 51, *p*= 0.15, *r*= 0.56
HI-OPPO		*T*= 82, *p*= 0.89, *r*= 0.35	*T*= 26 , *p*= 0.01*, *r*= 0.78
LO-BEND			*T*= 66, *p*= 0.3 *r*= 0.48
LO-OPPO			

The overall detection ratio (average of the performance in the four target locations) in the bends was lower in the steering task (76.8%, SD = 14.6%) vs. control task (81.7%, SD = 11.8%), which shows as a statistically significant difference from zero in within participant averaged detection ratio changes (Wilcoxon signed rank test *T* = 47, *p* =0.006, *r* = 0.66). We could not observe a statistically significant difference on the straights (steering task mean 81.6%, SD = 13%; control task mean 82.4%, SD = 13.6%; Wilcoxon signed rank test *T* = 117, *p* = 0.76).

### Reaction times to peripheral targets

**Figure 8:**
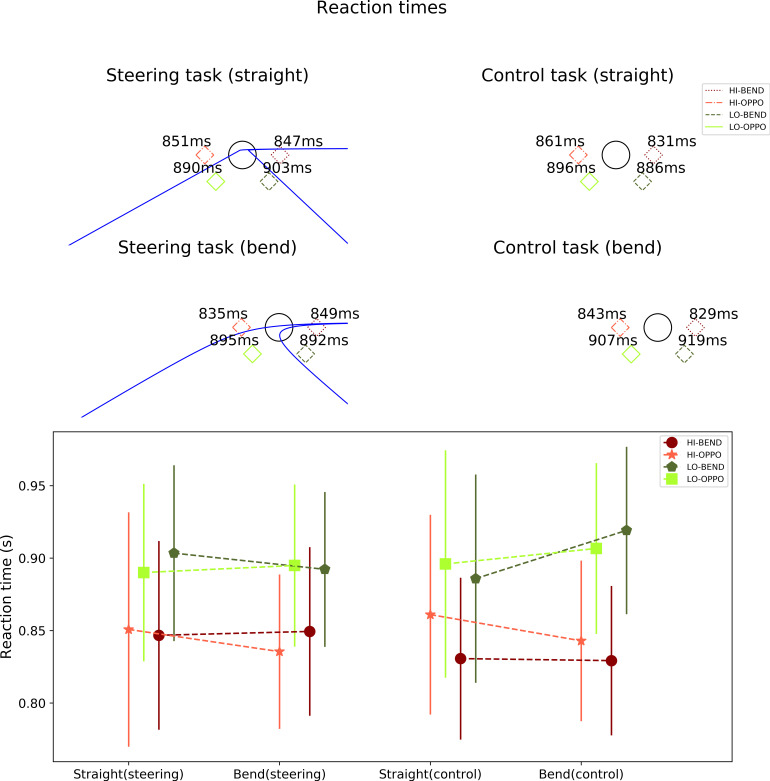
*Top*. Mean reaction times for targets presented in the different target locations, in the steering and control tasks and on the straights and in the bends. *Bottom*. The mean reaction times and standard deviations in a line plot.

Mean of participant median reaction times to target presentation was calculated for each target position, separately for the straights and the bends, and the steering and control tasks (Figure 8 and Table 4). Reaction time was only determined for (true) positive responses as there was no response for ‘non-detection’. Reaction time was determined from the beginning of the target presentation (which lasted for 0.5 s). Reaction time change was calculated by subtracting control task values from corresponding steering task values (Table 5).

**Table 4:** Mean reaction times (of participant medians) and standard deviations for the different target locations in bends and on straights in both the steering and control tasks.

Steering task (straight)	HI-BEND	HI-OPPO	LO-BEND	LO-OPPO
Mean	847.3 ms	850.8 ms	903.4 ms	890.0 ms
SD	129.8 ms	161.7 ms	121.4 ms	122.5 ms
Steering task (bend)	HI-BEND	HI-OPPO	LO-BEND	LO-OPPO
Mean	849.4 ms	835.5 ms	892.2 ms	894.9 ms
SD	116.4 ms	106.5 ms	106.9 ms	111.9 ms
Control task (straight)	HI-BEND	HI-OPPO	LO-BEND	LO-OPPO
Mean	830.7 ms	861.0 ms	885.8 ms	896.0 ms
SD	111.7 ms	137.8 ms	143.8 ms	156.9 ms
Control task (bend)	HI-BEND	HI-OPPO	LO-BEND	LO-OPPO

**Table 5:** Mean reaction time changes and standard deviations for the different target locations in straights and on bends.

Normalized (straight)	HI-BEND	HI-OPPO	LO-BEND	LO-OPPO
Mean	16.6 ms	-10.2 ms	17.6 ms	-5.9 ms
SD	91.6 ms	117.3 ms	90.4 ms	128.7 ms
Normalized (bend)	HI-BEND	HI-OPPO	LO-BEND	LO-OPPO
Mean	20.2 ms	-7.5 ms	-26.9 ms	-11.7 ms
SD	76.3 ms	67.2 ms	92.7 ms	82.0 ms

Reaction time changes were not significantly different across target positions in either of the two conditions (straights: Friedman test *Q* = 2.95, *p* = 0.40, in the bends: Friedman test *Q* = 6.76, *p* = 0.08), nor was there an overall difference in reaction times between the steering and control task (straights: Wilcoxon signed rank test *T* = 125, *p* = 0.69, in the bends: *T* = 123, *p* = 0.65). In both conditions, however, the HI targets were detected significantly faster than the LO targets (steering: Wilcoxon signed rank test *T* = 50, *p* = 0.007, *r* = 0.64; control: *T* = 40, *p* = 0.003, *r* = 0.71) with lower means for HI-BEND and HI-OPPO than for LO-BEND and LO-OPPO.

## Discussion

Eye tracking methods generally only inform about overt attention and provide limited grounds to assess hypotheses concerning covert attention. In laboratory tasks, it is possible to probe covert attention shifts by behavioural means, such as recording reaction times or discrimination ability for objects in the visual periphery. This study is an attempt to bring highly controlled laboratory paradigms closer to the perceptual-cognitive demands our visual system actually faces in natural dynamic tasks such as driving.

Inspired by the Posner cue paradigm, we investigated whether the detection of visual targets is facilitated by covert attention shifts “cued” by the visuomotor task of steering itself. According to the motor theory of attention, overt gaze shifts, on the one hand, are preceded by shifts in covert attention, and on the other hand, even in situations without overt saccades (covert) visuospatial attention shifts are at least in part dependent on the same saccade planning processes. Given that saccades to further ahead from the guiding fixation region (about 2 seconds into the future, directly ahead on the straights and in the direction of a bend while cornering) are a robust and probably highly automatic eye movement pattern for drivers, we asked whether saccade planning might show up as a covert attention effect even in a task where fixation is maintained under instruction within the guiding fixation region.

Also, it has been shown that visual flow in a naturalistic scene display biases attention in the direction of the focus of optical expansion (i.e. opposite the direction of flow lines). On a straight this would be symmetrical around the straight ahead/focus of expansion (but could bias attention towards the “far” region/ horizon). In bends, there is no focus of optical expansion per se, but self-rotation does produce a significant horizontal component to optic flow, which could show up as covert attentional (bottom-up) bias toward the direction of rotation. Top-down motor planning for steering and eye movements could produce a similar bias. We probed these issues by looking for facilitated peripheral target processing in visual locations in the direction of the upcoming road in an active visual steering task (extended sequences of locomotion over a textured terrain).

Comparing target detection performance in the steering tasks to detection of the same targets without the visuomotor component showed on average accuracy was impaired slightly by the active task during bends but not during the straight portions. This may be interpreted as the steering task “binding” attention to the current gaze position. But this must be considered a very general effect, and may not be very informative about underlying mechanisms. For example, the effect could be due to cognitive load imposed by increased task demand – cognitive workload imposed by secondary tasks such as N-back has been found to decrease performance at peripheral discrimination (31). And, keeping in mind the connection between attention and saccade planning, Lehtonen, Lappi and Summala (32) found that a non-visual working memory task (self-paced serial addition) reduced the number of look-ahead fixations in approaching a bend whereas Mars & Navarro (19) and Schnebelen et al. (33) found that removing the need for active steering (but keeping the visual stimulation constant) increased the number of look-ahead fixations. However, in the present experiment differences in performance in the two conditions can also depend stimulus differences in a more bottom-up way: e.g. due to masking/distraction by the complex road and optic flow stimulus, or other factors such as blurring of the stimulus due to presence of optokinetic nystagmus in the steering but not the control task.

Our more specific hypotheses that predicted specific patterns of asymmetry in peripheral target detection (viz. HI-BEND from look-ahead fixation planning, LO-BEND from peripheral road edge monitoring and LO-OPPO in bends from attention to the near future path) were not supported. Comparing the different target positions, we found that in straights the detection of targets presented in the lower target position opposite the (upcoming) bend direction was proportionately more impaired in the steering task than detection of the targets presented in the higher position in that direction. In the bends, the detection rates for the LO targets were lower than for the HI targets, but the differences did not reach statistical significance.

What this appears to indicate is that the “near” path (straight ahead of the vehicle) is not readily attended to in constant-radius curve driving. (One might propose that perhaps peripheral monitoring of the “near” road may be more directed to the road edge than a “near” future path location, but this should be observed as detection asymmetry in the low positions; detection ratios on the straights were LO-OPPO = 72.7% and LO-BEND = 80.5%, which did not reach significance). Another possible interpretation is that the head tilt (in the direction of the bend; 34) moves this target lower in the visual field, impairing detection.

In both conditions, the upper targets (at the same vertical level as the fixation circle) were detected faster than the lower targets, even though the visual eccentricity of all targets was similar. As most saccades in natural behaviour are horizontal, this can be considered tentative support for an oculomotor planning mechanism being involved. However, this conclusion must be tempered by the fact that the a priori predicted asymmetry between upper targets (in the direction of the bend vs. opposite) was not observed. That is, the processing of targets that appeared in the look-ahead fixation location was not facilitated in this peripheral pattern detection task.

### Limitations of the study

The Posner paradigm probes selective attention with a pattern discrimination task. It may be argued that this is not representative of the ecological demands on peripheral attention in driving tasks. Indeed, a multiple object tracking task could better capture the attentional processes most relevant to safe and efficient steering. The present experiment may be considered more analogous to the task of peripherally detecting important symbolic information in instruments or a Head-Up Display.

The study was conducted in a driving simulator where the road scene was rendered on a 2D display. Therefore, the third, depth, dimension of actual steering tasks was ignored. If saccade planning (and by the premotor theory covert attention) takes place in 3D coordinates, then an immersive 3D VR environment would be more appropriate to study the phenomenon.

Inhibition of return can complicate the picture with respect to reaction times if attention is not distributed in *parallel* but instead acts as a “spotlight” that *serially* visits the target locations. Posner and Cohen (35) observed that cues made reaction times faster when the target was presented closely after the cue, but if the target was presented >300 ms after the cue response times were made longer. This is interpreted as attention visiting the target location and then withdrawing subsequently suppressing the processing of the visited location. This potentially is a problem for probing covert attention shifts in a continuous dynamic task where the “cue” is present all the time, and it is not known precisely when the attention might have shifted to the periphery, and when it might have shifted back. Designs where more discrete “events” can be identified in the steering task might in the future elucidate these matters.

### Conclusions and future directions

In complex dynamical tasks such as in driving not is it essential to direct gaze in the appropriate location in space at the appropriate time, but covert attention needs to be adaptively distributed in the visual periphery as well. Eye tracking in naturalistic and simulated driving tasks have provided a great deal of information about drivers’ visual behaviour and gaze strategies but provide limited grounds to assess hypotheses concerning covert attention. This study shows that active steering and rich visual stimuli in a temporally extended natural steering task do affect the distribution of covert attention, as indexed by peripheral target detection. However, none of our more specific hypotheses derived from the previous literature was supported by the pattern of the detection data.

To understand covert attention in such tasks it is desirable to further develop experimental paradigms such as the present one, which will allow more complex stimuli and natural, extended task sequences to be used; the visual world does not appear to us as a sequence of “trials” where stimuli are “presented” and “responded” to in a discrete manner. Attention, motor control and perception are intertwined, and teasing apart underlying mechanisms is a challenge. Rising to this challenge requires clever experimental designs that can combine ideas and techniques from restricted laboratory studies with more naturalistic task constraints. Thus, gradually, a more ecologically valid yet rigorously founded view of the role of attention in dynamic natural tasks should emerge.

## Ethics and Conflict of Interest

All participants signed an informed consent form and approved the use and publication of the data for scientific purposes. The study was approved by the University of Helsinki ethics committee and followed the declaration of Helsinki and guidelines of the Finnish ethical board (www.tenk.fi). The authors have no conflicts of interests to declare.
